# Accelerometry-derived physical activity patterns and clinical correlates in Swiss geriatric inpatients: a cross-sectional study

**DOI:** 10.1186/s12877-026-08064-8

**Published:** 2026-08-04

**Authors:** Caroline Yvonne Tanner, Frank Stoller, Miriam Gähwiler, Livia Vinzens, Dzuza Papik, Jessica Scherz, Patrick Eggenberger, Martina Betschart

**Affiliations:** 1 Department of Health, Physiotherapy, OST Eastern Switzerland University of Applied Sciences, St. Gallen, Switzerland; 2 Department of Health Sciences and Technology, Institute of Human Movement Sciences and Sport, ETH Zurich, Zurich, Switzerland; 3 Geriatrische Klinik St. Gallen AG, St.Gallen, Switzerland; 4 Kantonsspital Winterthur, Geriatric Clinic, Winterthur, Switzerland

**Keywords:** Accelerometry, Wearable sensors, Physical activity, Upright time, Sedentary behavior, Locomotor capacity, Hospitalized older adults, Acute geriatric care

## Abstract

**Background:**

Physical activity among hospitalized geriatric patients is very low, contributing to functional decline and delayed recovery. However, posture-sensitive thigh-worn accelerometry has not yet been used to assess physical activity and its clinical correlates in Swiss acute geriatric inpatients. This multicenter cross-sectional study aimed to (a) quantify physical activity patterns and (b) examine associations with demographic and clinical characteristics.

**Methods:**

Inpatients from two acute geriatric care hospitals wore activPAL accelerometers for up to 7 consecutive days. The device recorded the duration of specific activity patterns, including walking, standing, sitting, and lying as well as step count and cadence. Demographic and clinical characteristics, including sex, age, locomotor capacity (de Morton Mobility Index, DEMMI), cognitive function (Mini-Mental State Examination, MMSE), comorbidity burden (Charlson Comorbidity Index, CCI), independence in basic activities of daily living (Barthel Index, BI), gait capacity (10-Meter Walk Test, 10-MWT), and lower limb strength (hand-held dynamometry), were examined for their associations with upright time (i.e., standing and walking) using multiple linear regression.

**Results:**

Eighty-nine inpatients (mean age 83.7 ± 6.9 years; range 62–97 years; 64.0% women) were included in the final analysis. Upright time accounted for 12.4% of awake time (110.7 ± 74.8 min). Patients spent 2.1% of awake time walking, 10.3% standing, 75.5% sitting, and 12.0% lying. Walking corresponded to 19.0 ± 14.0 min/day and 1,278.7 ± 1,047.3 steps/day. The regression model explained 28.9% of the variance in upright time, with DEMMI, CCI, and knee extension strength showing the strongest associations.

**Conclusions:**

Acute geriatric inpatients were largely inactive, showing fragmented walking and prolonged sitting bouts. Simple assessments of locomotor capacity, comorbidity burden, and lower-limb strength may support stratification of geriatric inpatients according to their functional capacity and help inform individualized physical activity prescriptions.

## Background

Ageing is inevitably associated with declines in physical and cognitive capacities, which in turn are linked to increased mortality, as well as higher rates of hospitalization and rehospitalization [1, 2]. Nevertheless, physical activity is one of the most effective and modifiable strategies to counteract the risk of age-related disease. In older adults, regular moderate to high levels of physical activity are associated with a 26–35% lower risk of cognitive decline, a 14–21% lower risk of developing dementia, and a 17% lower risk of depressive symptoms. In addition, physical activity has been shown to prevent falls and improve functional ability [3]. In Switzerland, 28% of adults aged over 65 years are insufficiently physically active [4].

Hospitalization has been shown to exacerbate physical inactivity, with hospitalized geriatric patients standing and walking approximately 2.4 times less than individuals receiving care at home [5]. Systematic reviews involving adults aged over 60 years found that physical activity during hospitalization is associated with better health outcomes, including reduced pain, delirium, risk of falls, depression, and complications such as deep vein thrombosis and cardiovascular events, as well as improved cognition and quality of life [6–10]. Despite this, research indicates that acute geriatric inpatients spend 83% to 89.2% of the day sedentary, as assessed by waist- or thigh-worn accelerometry [9, 11, 12].

A study conducted in a Swiss geriatric ward confirmed these low physical activity levels using wrist-worn accelerometry. The authors reported 90.4% inactivity per day and found that fewer than 10% of inpatients reached ≥20 min of moderate-intensity physical activity per day [13]. In a secondary analysis of the same cohort, a median of 603 steps per day was observed, with only 3% of inpatients walking continuously for ≥2 min per day [14]. This is clinically relevant because walking fewer than 900 steps per day has been associated with a substantially increased risk of hospitalization-related functional decline [15]. Nonetheless, data on physical activity patterns in hospitalized older adults in Switzerland and other German-speaking countries remain limited. In particular, no prior studies in Swiss acute geriatric inpatients have used posture-sensitive thigh-worn accelerometry to assess physical activity and its clinical correlates. Thigh-worn sensors are considered highly valid and better suited than wrist-worn accelerometers for posture detection [16, 17]. Improved understanding of the clinical correlates and determinants of physical activity may help characterize physical activity patterns in acute geriatric inpatients and support targeted promotion of physical activity and healthy lifestyles. Routine assessments may also serve as practical indicators of patients’ functional capacity. This information can help healthcare professionals tailor individualized physical activity prescriptions regarding type, intensity, frequency, and supervision [18]. For example, a patient who spends most waking hours sedentary and presents with low locomotor capacity, high comorbidity burden, and reduced muscle strength may initially benefit most from reducing sedentary behavior and engaging in light-intensity physical activity. In contrast, older adults who are already more physically active may require higher-intensity or more progressive activity stimuli to achieve further functional improvements and health benefits [19].

Therefore, this study aimed to (a) quantify physical activity patterns using thigh-worn accelerometry and (b) assess associations with demographic and clinical characteristics in Swiss acute geriatric inpatients. We hypothesized that upright time (i.e., standing and walking) would (a) account for less than 20% of awake time, based on prior inpatient accelerometry studies [5, 9, 12] and (b) be associated with sex, age, locomotor capacity, cognition, comorbidity burden, independence in basic activities of daily life (ADL), gait capacity, and lower limb strength.

## Methods

### Study setting and design

This multicenter cross-sectional study was conducted between February and June 2024 in the acute geriatric wards of Kantonsspital Winterthur and Geriatrische Klinik St. Gallen in Switzerland. Patients typically remain on the ward for 7 to 14 days, with length of stay being predetermined by the Swiss Diagnosis-Related Groups (DRG) system in acute geriatric care. Patients received at least two therapeutic interventions per day, each lasting 30–60 min, as defined by the Swiss Classification of Surgical Interventions (CHOP). These interventions included physical and occupational therapy; if indicated, patients also received speech or dysphagia therapy. The study was approved by the local Ethics Committee of Eastern Switzerland (EKOS, project number 2023–02103). All patients provided written informed consent before study participation. The study was conducted in accordance with the Declaration of Helsinki, and reporting followed the Strengthening the Reporting of Observational Studies in Epidemiology (STROBE) guideline. As an observational cross-sectional study, it was not prospectively registered.

### Patient recruitment

At each study site, a qualified physiotherapist (D.P. and J.S., respectively) screened patients admitted to the acute geriatric wards for study eligibility. Patients were eligible if they were expected to remain hospitalized for at least 7 days and had provided informed consent. Patients were eligible if they were able to walk with or without personal assistance and/or assistive devices (e.g., walkers). Individuals were excluded if they had delirium or confusion, a planned transfer to a palliative care or intensive care unit, insufficient German language skills, placement in an isolation room, and/or an inability to understand instructions. Delirium and disorientation were defined as a score of ≥ 3 on the Delirium Observation Screening Scale (DOS) [20, 21] or > 2 on the Nurse-Based Delirium-Screening Tool (NU-DESC) [22]. These validated screening tools were administered by trained nurses as part of standard care assessments.

### Accelerometry-derived physical activity assessment

**Fig. 1:**
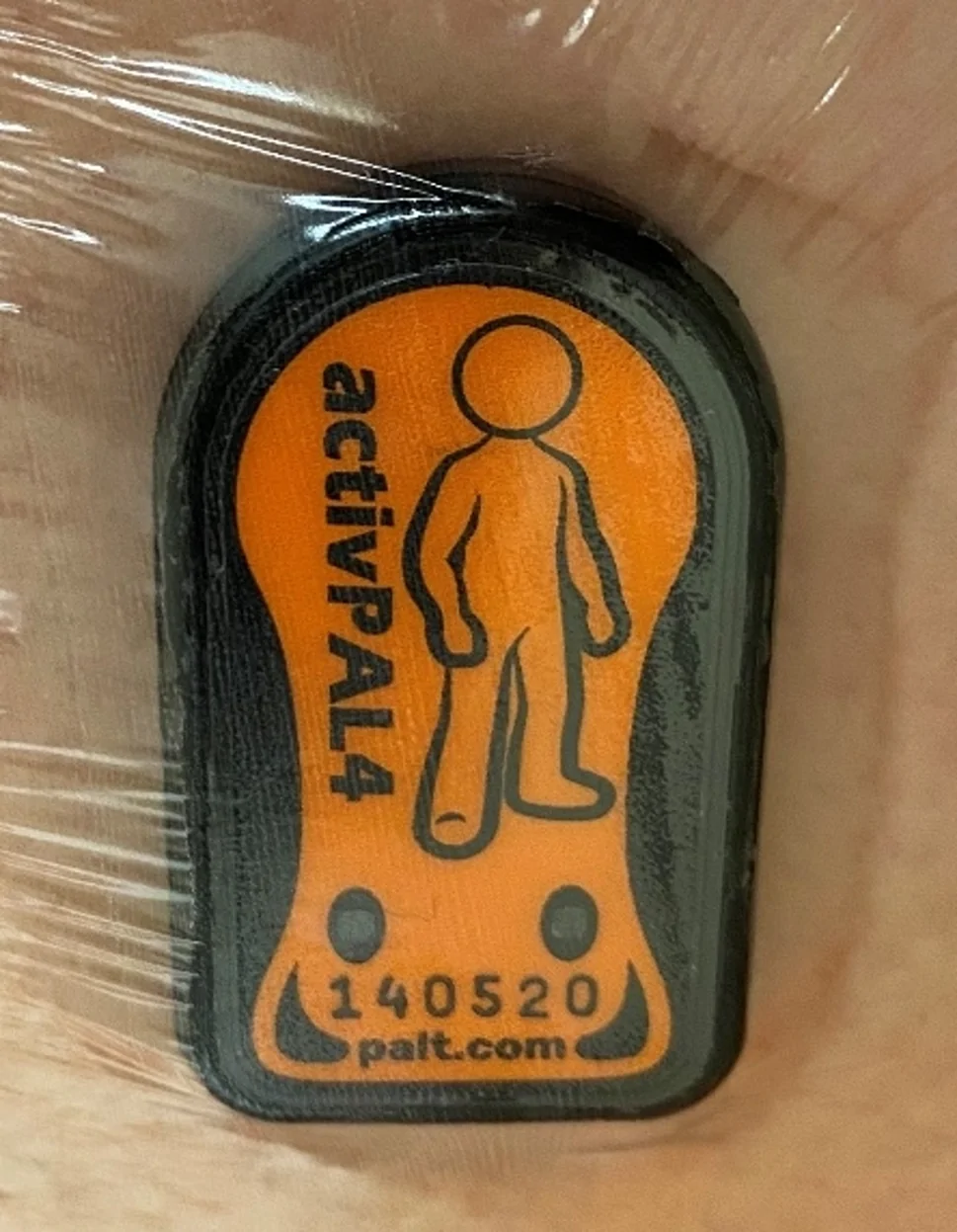
activPAL sensor used for physical activity assessment, attached to the anterior thigh 15 cm above the patella

Physical activity patterns of patients were assessed using the thigh-worn activPAL accelerometer/inclinometer (PAL Technologies Ltd., Glasgow, United Kingdom; Fig. 1). Data were processed using PALanalysis software (version 8.11.8.75) with the MORA validation algorithm and the CREA 1.3 m classification algorithm. The activPAL has been validated and is frequently used to objectively quantify physical activity in older adults [23, 24].

Based on their reported relevance for locomotor capacity and functional status in hospitalized geriatric patients, the primary accelerometry-derived parameters were total upright time (i.e., standing and walking), total walking time, total sitting time, primary lying time (i.e., nighttime), secondary lying time (i.e., daytime), and step count [15, 25, 26]. Physical activity distribution was calculated as a percentage of awake time, which was defined as the 24-hour day minus primary lying time. Additional clinically informative parameters included bout-based characteristics of walking and sitting, cadence, and the number of sit-to-stand transitions [14, 25, 27]. For readability, the activPAL-derived stepping category is referred to as walking throughout the manuscript.

The sensor was attached 15 cm above the patella using transparent, skin-friendly waterproof tape (Opsite Flexfix, Smith & Nephew) and was worn continuously for up to 7 days with a sampling frequency of 20 Hz. Physical activity monitoring was initiated immediately after completion of the demographic and clinical assessments within the first two days after admission. Healthcare staff temporarily removed and replaced sensors during radiation procedures (e.g., CT scans), and all off-wear periods were logged and verified against the recorded data. A measurement day was considered valid if at least 20 h of data were available after excluding non-wear periods. The first and last measurement days were excluded because they typically did not provide sufficient recording time to meet the valid day criteria, resulting in a maximum of 5 analyzable days. Patients were required to provide at least 3 valid days for inclusion in the analyses, consistent with the commonly recommended wear duration of 3–7 days [28–31]. Since physical activity may change during hospitalization, our analyses were limited to the first hospital week across all included patients. Outcome variables were calculated as the mean across all valid measurement days.

### Demographic and clinical characteristics

Demographic characteristics, including age, sex, and weight, were extracted from the hospital information system. Basic gait ability and the use of assistive walking devices relevant to the inclusion criteria were also retrieved and verified on site by the study team. In addition, clinical assessment data collected within the first two days after admission as part of routine hospital procedures were obtained. Locomotor capacity was quantified by trained physiotherapists or occupational therapists using the de Morton Mobility Index (DEMMI) [32, 33], and cognitive function was assessed using the Mini*-*Mental State Examination (MMSE) [34]. Comorbidity burden was calculated by the study team using the Charlson Comorbidity Index (CCI) [35], based on diagnoses retrieved from the hospital information system. The CCI includes 17 comorbidities weighted from 1 to 6 according to disease severity and mortality risk. ADL independence was assessed by trained nurses using the Barthel Index (BI). The BI is a validated standard assessment in nursing care [36, 37], with a total score of 0–20 indicating total dependence, 21–60 severe dependence, 61–90 moderate dependence, and 91–99 slight dependence, while a score of 100 indicates complete independence without the need for assistance from healthcare professionals. Gait speed and cadence were assessed using the 10-meter walk test (10-MWT), performed with assistive devices as required [38], and lower limb strength was measured using the MTAS F-1 handheld dynamometer (ANIMA Corporation). These measurements were performed before the activPAL sensor was attached. Knee extension and hip flexion strength were assessed in the seated position, whereas hip extension strength was measured in either the prone or semi-prone position [39]. Force values were recorded in kilogram-force, convert into Newtons (N) by multiplication with 9.80665, and then normalized to bodyweight. All assessment were conducted within the first two days after admission and before the activPAL sensor was attached.

### Statistics

Demographic, clinical, and physical activity data were summarized for the total sample and stratified by sex for descriptive purposes, given the established influence of sex on gait, locomotor capacity, muscle strength, physical activity patterns, and functional performance in older adults [40–43].

Exploratory multiple linear regression was performed to examine associations between upright time and eight predictors: age, sex, DEMMI, BI, CCI, MMSE, gait speed, and knee extension strength. Predictors were preselected based on theoretical and clinical relevance, prior evidence, data completeness, and avoidance of redundancy to minimize the risk of overfitting. Missing values were observed for DEMMI (4.5%), MMSE (4.5%), gait speed (21.3%), and knee extension strength (18.0%) and were addressed using multiple imputation by chained equations (MICE; m = 20; predictive mean matching), resulting in 20 fully imputed datasets for all 89 patients. Missing values in gait and lower-limb strength measures were mainly related to medical contraindications such as acute fractures or recent surgery. The regression model was fitted separately in each imputed dataset, and parameter estimates, standard errors, confidence intervals, and *p*-values were pooled using Rubin’s rules. Overall model significance was assessed using the D1 model comparison test for multiply imputed data. Multicollinearity was evaluated using variance inflation factors (VIFs). Hospital site was neither included as a covariate nor analyzed separately because of the limited sample size and the similar ward settings at both sites. Regression coefficients are reported with 95% confidence intervals and *p*-values, and model fit was summarized using pooled *R²*.

For descriptive comparisons between women and men, homogeneity of variance was assessed using Levene’s test, and normality of continuous variables was evaluated using the Shapiro–Wilk test and quantile–quantile plots. Depending on these assumptions, independent-samples *t*-tests, Mann–Whitney *U* Tests, Fisher’s exact tests, or *χ²* tests were used.

Statistical analyses were conducted in R (version 4.5.3) using RStudio (version 2026.01.2 + 418). Relevant analyses and visualizations were performed with the packages *car*,* mice*,* lm.beta*, and *ggplot2*. The significance level was set at *α* = 0.05, and effect sizes were interpreted as small for *r* = 0.10, moderate for *r* = 0.30, and large for *r* ≥ 0.50 [31].

## Results

### Demographic and clinical characteristics

**Fig. 2:**
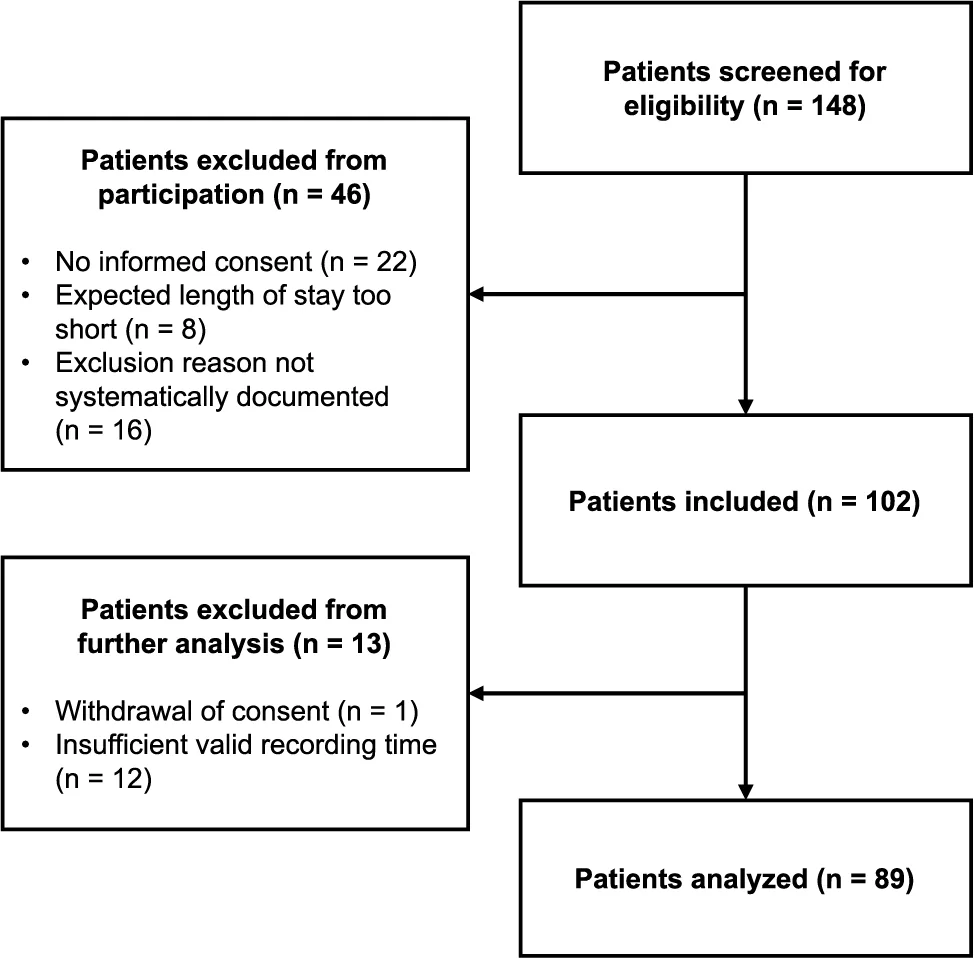
Patient flow diagram

Of the 148 patients screened for eligibility, 102 were included in the study. After exclusion of 13 patients from further analysis, the final analytical sample comprised 89 patients (57 women, 32 men; age range 62–97 years). Details of patient flow are shown in Fig. 2. Demographic and clinical characteristics are summarized in Table 1 for the overall sample and stratified by sex for descriptive purposes.

**Table 1:** Demographic and clinical characteristics

Variables	Overall	Female	Male	*p*
Demographic characteristics				
^ #^Sex / *N* (*n*, %)	89 (100%)	57 (64.0%)	32 (36.0%)	–
^ #^Age, years (mean, SD)	83.7 (6.9)	82.6 (7.0)	85.5 (6.4)	0.049*
Weight, kg (mean, SD)	67.2 (12.5)	65.4 (13.0)	70.4 (11.1)	0.039*
Clinical assessments				
^ #^DEMMI, score/100 (mean, SD)	46.5 (16.2)	48.1 (16.6)	43.6 (15.5)	0.220
^ #^MMSE, score/30 (mean, SD)	25.5 (3.7)	26.2 (3.8)	24.4 (3.3)	0.004*
^ #^CCI, score/6 (mean, SD)	4.4 (2.3)	4.0 (2.2)	5.1 (2.2)	0.017*
^ #^BI, score/100 (mean, SD)	74.9 (23.5)	80.6 (20.5)	64.6 (25.2)	< 0.001*
Gait capacity				
^ #^Gait speed 10-MWT, m/s (mean, SD)	0.68 (0.20)	0.70 (0.22)	0.64 (0.16)	0.074
Cadence 10-MWT, steps/min (mean, SD)	92.9 (16.4)	93.8 (16.6)	91.4 (16.1)	0.371
Lower limb strength				
^ #^Knee Extension, N/kg (mean, SD)	2.5 (1.0)	2.4 (1.0)	2.5 (1.2)	0.800
Hip Flexion, N/kg (mean, SD)	1.8 (0.8)	1.7 (0.7)	1.9 (0.9)	0.599
Hip Extension, N/kg (mean, SD)	1.6 (0.9)	1.4 (0.7)	2.1 (1.0)	0.018*

*Abbreviations*: *10-MWT* 10-Meter Walk Test, *BI* Barthel Index, *CCI* Charlson Comorbidity Index, *DEMMI* De Morton Mobility Index, *MMSE* Mini-Mental State Examination, *p p*-value, *SD* Standard deviation

*P*-values were calculated using either independent-samples *t*-tests, Mann–Whitney *U* Tests, Fisher’s exact tests, or *χ²* tests

* Indicates statistically significant sex differences; # indicates variables included as predictors in the regression analysis

### Accelerometry-derived physical activity patterns

**Fig. 3:**
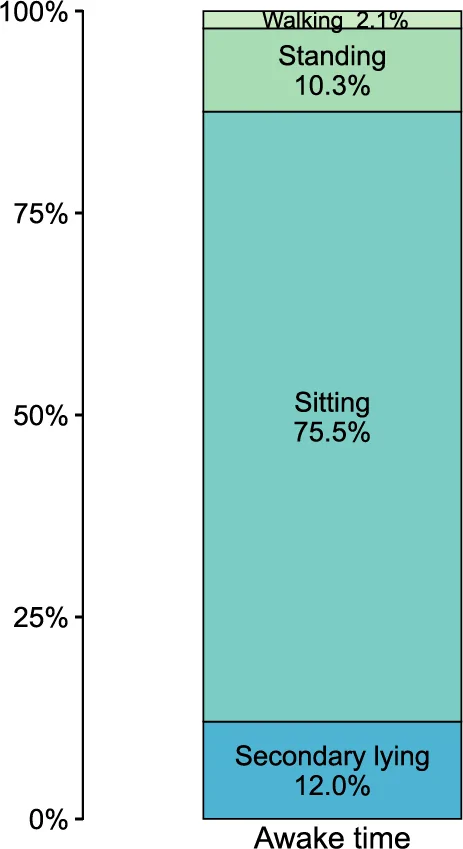
Percentage of awake time spent in different activities

**Fig. 4:**
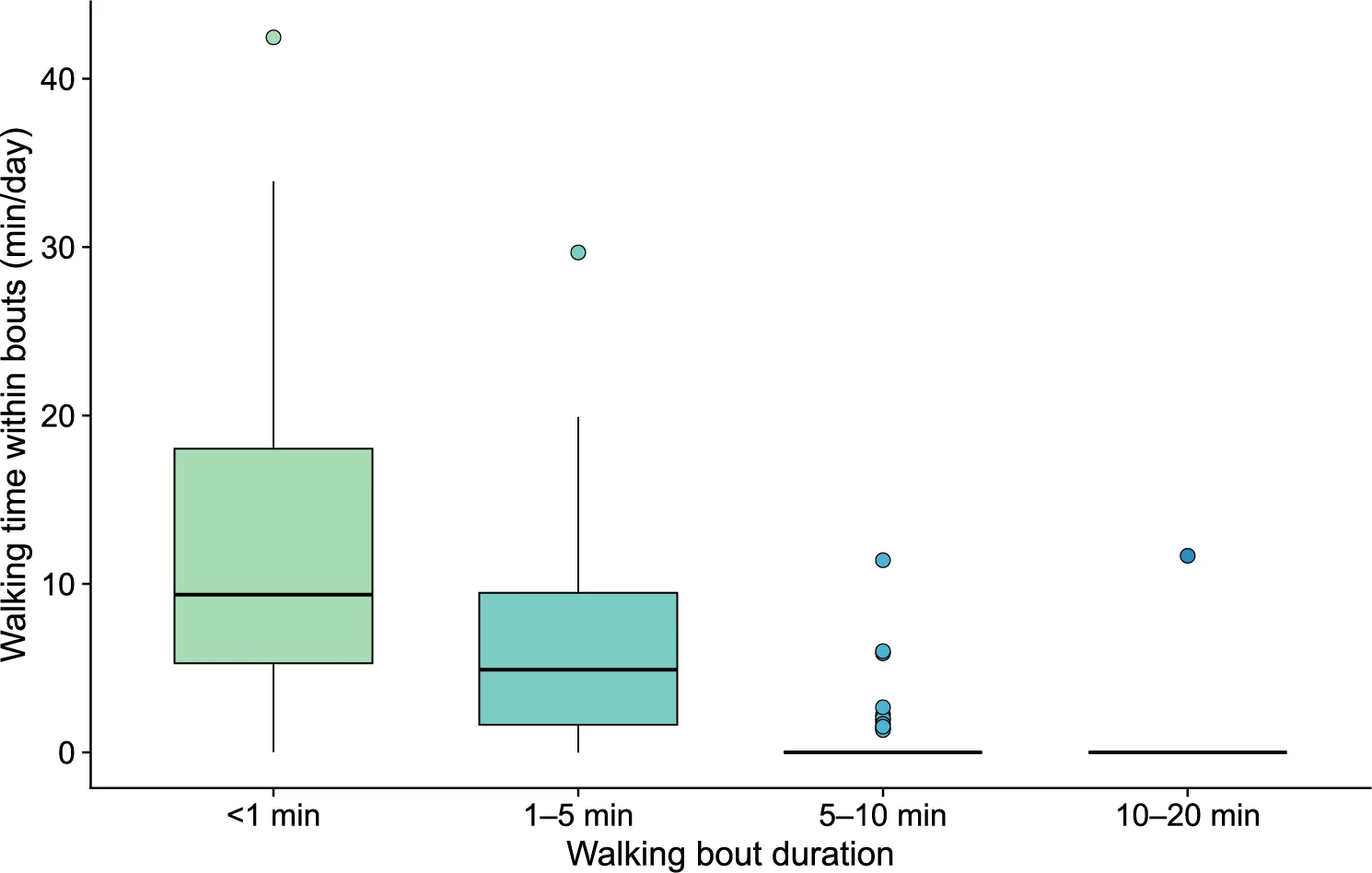
Distribution of walking time within continuous walking bouts

Physical activity variables derived from up to 7 days of thigh-worn accelerometry during the first week of hospitalization are presented in Table 2. In total, 348 valid monitoring days were included, corresponding to a mean of 3.9 ± 0.4 valid days per patient (2.5 weekdays and 1.4 weekend days) and an average wear time of 1439.0 ± 11.4 min/day. During awake time, total upright time (i.e., standing and walking) accounted for 12.4%, total sitting time for 75.5%, and secondary lying time for 12.0% (Fig. 3). On average, patients spent 19.0 ± 14.0 min per day walking and accumulated 1,278.7 ± 1,047.3 steps per day. Total walking time was further subdivided into continuous walking bouts of different durations to characterize fragmented versus sustained walking (Fig. 4). No statistically significant differences were found between women and men.

**Table 2:** Average 24-hour physical activity patterns

Variables	Overall	Female	Male	*p*
Standing and Walking				
^ #^Total upright time (standing and walking), min (mean, SD)	110.7 (74.8)	122.2 (81.0)	90.2 (57.9)	0.067
Total walking time, min (mean, SD)	19.0 (14.0)	20.3 (15.1)	16.5 (11.5)	0.336
Total steps, *n* (mean, SD)	1,278.7 (1047.3)	1,382.7 (1135.2)	1,093.4 (854.5)	0.345
Steps at cadence 75–100 steps/min, *n* (mean, SD)	578.5 (706.6)	654.7 (763.0)	442.6 (579.4)	0.421
Steps at cadence > 100 steps/min, *n* (mean, SD)	70.9 (181.6)	80.6 (176.7)	53.6 (191.7)	0.377
Sitting				
Total sitting time, min (mean, SD)	679.9 (196.5)	690.4 (186.2)	661.2 (215.4)	0.888
Sitting time within bouts of > 60 min, min (mean, SD)	393.2 (241.6)	391.9 (238.6)	395.5 (250.6)	0.884
Number of sitting bouts lasting > 60 min, *n* (mean, SD)	3.0 (1.8)	3.1 (1.7)	2.8 (1.8)	0.509
Sit-to-stand-transitions, *n* (mean, SD)	36.7 (18.2)	38.2 (17.2)	34.0 (19.7)	0.122
Lying				
Primary lying time (nighttime), min (mean, SD)	528.9 (147.4)	523.8 (139.5)	538.1 (162.6)	0.434
Secondary lying time (daytime), min (mean, SD)	104.8 (133.3)	96.3 (126.7)	119.8 (145.1)	0.263

*Abbreviations*: *p p*-value, *SD* Standard deviation

*P*-values for female versus male comparisons were calculated using either independent-samples *t*-tests, Mann–Whitney *U* Tests, Fisher’s exact tests, or *χ²* tests

# Indicates the dependent variable in the regression analysis

### Demographic and clinical correlates of upright time

The pooled multiple imputation regression model explained 28.9% of the variance in upright time (*R²* = 0.289, 95% CI 0.136 to 0.451). The overall pooled model was significant according to the D1 model comparison test (*F*(8, 77.82) = 3.84, *p* < 0.001). Multicollinearity was low across imputed datasets, with VIFs ranging from 1.14 to 1.68 for individual predictors. Pooled regression coefficients for all predictors are presented in Table 3.

**Table 3:** Pooled regression model coefficients

Predictors	B (95% CI)	SE	*p*	β
DEMMI	1.5 (0.5 to 2.6)	0.527	0.006*	0.334
CCI	-7.2 (-13.9 to -0.5)	3.355	0.035*	-0.220
Knee extension strength	14.8 (-1.3 to 31.0)	8.112	0.072	0.204
Sex	-13.6 (-47.3 to 20.1)	16.919	0.424	-0.182
MMSE	2.2 (-2.1 to 6.4)	2.133	0.311	0.111
Age	0.5 (-1.7 to 2. 7)	1.095	0.657	0.045
BI	0.1 (-0.6 to 0.9)	0.393	0.721	0.043
Gait speed 10-MWT	-14.0 (-96.6 to 68.6)	41.383	0.736	-0.039

*Abbreviations*: *10-MWT* 10-Meter Walk Test, *B* Unstandardized regression coefficient, *β* Standardized regression coefficient, *BI* Barthel Index, *CCI* Charlson Comorbidity Index, *CI* confidence interval, *DEMMI* De Morton Mobility Index, SE Standard error, *MMSE* Mini-Mental State Examination, *p p*-value

* Indicates statistical significance. Predictors are sorted by the absolute standardized *β* coefficient. Female was the reference category for sex

## Discussion

To our knowledge, this is the first study to investigate detailed physical activity patterns and their clinical correlates in Swiss acute geriatric inpatients using thigh-worn accelerometry. The main findings were that (a) patients in Swiss acute geriatric hospitals were largely physically inactive, spending only 12.4% of awake time in upright activity, corresponding to 91.7 min of standing and 19.0 min of walking, and (b) locomotor capacity, comorbidity burden (CCI), and lower-extremity strength showed the strongest associations with upright time.

### Accelerometry-derived physical activity patterns

In line with the present study, previous research in hospitalized older adults reported similarly low upright times ranging from 102 min [12] to 122 min [5], with one study reporting as little as 43 min [11]. A longitudinal study in acutely hospitalized older adults reported increases in upright time alongside concomitant reductions in sedentary behavior from hospitalization to post-discharge. As the present study was limited to the first week of hospitalization, no conclusions can be drawn regarding temporal changes in physical activity patterns over the course of the hospital stay [12].

The mean daily step count of 1,278.7 observed in our study was comparable to that reported in geriatric rehabilitation inpatients (1,236 steps) [5]. However, our cohort showed higher walking volumes than other acute geriatric inpatient groups. For example, a Swiss study conducted in the French-speaking part of Switzerland reported 603 steps per day [14], and a Danish study reported 728 steps per day [12]. These differences in step count may be explained by the inclusion of more vulnerable patients in the latter two studies, particularly patients with greater cognitive impairment. Impaired cognition has previously been associated with lower odds of achieving recommended physical activity levels [44].

Walking patterns were highly fragmented, with most of the total daily 19.0 min of walking accumulated in very short bouts of less than 1 min and 1 to 5 min (Fig. 4). This finding is consistent with other studies in hospitalized geriatric patients, which reported that walking occurred mainly in bouts of less than 30 s [14]. Approximately half of the daily accumulated steps were performed at a low cadence of < 75 steps/min, whereas during the 10-MWT at individually preferred walking speed, 75% of patients achieved a substantially higher cadence ranging from 84 to 107 steps/min. Furthermore, patients demonstrated moderately reduced locomotor capacity as reflected by the mean DEMMI score, and none had a formal prescription for bed rest. This apparent capacity–performance gap suggests that the low activity levels observed cannot be explained by severe locomotor limitation alone. Rather, functional ability does not necessarily translate into real-world locomotor behavior in the acute geriatric hospital setting, likely because of additional barriers to physical activity such as fatigue, concerns about falling, medical devices, supervision needs, and environmental constraints.

Pronounced sedentary behavior in hospitalized older adults has been reported in several studies [45]. In line with this, our cohort spent a large proportion of awake time sitting (75.5%; 679.9 min). Importantly, 58% of total sitting time (393.2 min) was accumulated, on average, in three prolonged bouts lasting more than 60 min. This is clinically relevant because prolonged uninterrupted sitting bouts ≥60 min during hospitalization have been associated with poorer ADL recovery in older adults, independently of total sedentary time [46].

### Demographic and clinical correlates of upright time

In our exploratory regression analysis, demographic and clinical characteristics moderately explained variation in upright time (*R*^*2*^ = 28.9%), comparable to previous inpatient models explaining approximately 28–43.1% of the variance in accelerometry-derived physical activity [26, 47, 48]. Based on the magnitude of their standardized *β* coefficients and statistical significance, locomotor capacity (DEMMI) and comorbidity burden (CCI) emerged as the strongest independent predictors of upright time. Knee extension strength also showed a similarly large standardized *β* coefficient, suggesting a possible association, although the estimate remained non-significant. In practical terms, assuming the other model variables remain constant, a 10-point higher DEMMI score would correspond to approximately 15 additional minutes of upright time per day, whereas a 2-point higher CCI score would be associated with an estimated 14.4-minute decrease. In addition, a 1 N/kg higher knee extension strength would correspond to an estimated increase of 14.8 min.

To our knowledge, knee extension strength has not yet been investigated as a predictor of physical activity in hospitalized older adults. However, previous studies support associations of locomotor capacity (DEMMI) [47, 48], comorbidity burden (CCI) assessed with a comparable morbidity measure [49], and lower-extremity functioning as assessed by the Short Physical Performance Battery (SPPB) [26] with upright time in this population. Although isolated knee extension strength is not equivalent to the SPPB, this suggests that lower-extremity capacity is relevant for upright physical activity. In addition, knee extension strength is sensitive to deconditioning during hospitalization [50] and improvements in knee extension strength have been accompanied by increased daily step count in post-hospitalization rehabilitation programs [51]. This supports the view that our findings are clinically meaningful rather than incidental. Sex, age, cognitive function (MMSE), ADL independence (BI), and gait speed remained non-significant in the regression model, and their standardized *β* coefficients were smaller, indicating that their associations with upright time were weaker than those of DEMMI, CCI, and knee extension strength.

### Implications for clinical practice

Promotion of physical activity appears essential for all geriatric inpatients. Nonetheless, based on our finding that lower DEMMI scores and higher CCI scores were associated with reduced upright time, particular attention should be given to patients with lower locomotor capacity (DEMMI) and higher comorbidity burden (CCI). This may include the provision of appropriate walking aids and support from healthcare staff, as well as regular review and removal of urinary catheters and infusions, where medically feasible, to facilitate locomotion and increase freedom of movement [52]. In this context, assessments of DEMMI, CCI, and potentially knee extension strength at hospital admission may support stratification of geriatric inpatients according to their functional capacity and help guide individualized physical activity prescriptions.

The capacity–performance gap observed in our study may also be related to insufficient patient education and motivation. A recent systematic review highlighted the central importance of educational measures for promoting physical activity [53]. This underlines the importance of informing and training nursing staff, patients, and their relatives about the role of physical activity for recovery. Moreover, lack of patient motivation, time constraints among healthcare staff, and limited financial resources in hospitals appear to hinder physical activity in hospitalized older adults [54]. Resource-efficient interprofessional programs that encourage movement and reduce sitting time could help address this issue [54]. For example, frequent interruption of the prolonged sitting bouts observed in our cohort may represent a simple intervention to support functional recovery in older adults [46] and has been associated with improved metabolic health in non-hospitalized older adults [55]. Further potentially relevant interventions may include additional walking and simple multicomponent exercise sessions integrated into routine care [56, 57].

### Strengths and limitations

This study has several methodological strengths. These include the multicenter design with a relatively large number of patients and the objective quantification of physical activity using highly valid thigh-worn sensors, which minimized subjective bias and increased the reliability of data collection. Thigh-worn sensors are advantageous over wrist-worn sensors for detecting upright postures [16, 17] and were therefore well suited for measuring physical activity in our cohort of geriatric inpatients who were able to walk. However, in studies including patients who are unable to walk, wrist-worn sensors may provide complementary information on upper-limb movement [58].

Nonetheless, a main limitation of the study is the exclusion of patients with moderate or severe cognitive deficits, delirium, non-German-speaking backgrounds, and those with irregular accelerometry patterns. As such, the most vulnerable patients have likely been removed from the sample, which may have resulted in an overestimation of physical activity levels of the broader acute geriatric inpatient population. Furthermore, the limited time available for recruitment and informed consent procedures meant that informed consent from legal guardians or relatives could not always be obtained in time. This may have also contributed to the exclusion of particularly vulnerable patients. Since our measurements were restricted to the first hospital week, the findings of this study primarily reflect early inpatient activity. Another limitation includes that no standardized logbook was maintained to document specific therapeutic interventions, making it impossible to distinguish between self-initiated and therapy-driven physical activity.

## Conclusion

Our findings show that older inpatients in Swiss acute geriatric hospitals spent only 12.4% of their awake time in upright activity, walked an average of 19.0 min and accumulated 1,278.7 steps per day. Since locomotor capacity (DEMMI), comorbidity burden (CCI), and knee extension strength showed the strongest associations with upright time, these simple measures may help stratify geriatric inpatients according to their functional capacity and guide individualized physical activity prescriptions. Although many patients demonstrated the capacity for more intensive walking during formal testing, their actual daily physical activity remained low. This underscores the potential to promote physical activity through education and structured activity plans beyond therapy sessions.

## Data Availability

The datasets used and/or analyzed during the current study are available from the corresponding author on reasonable request.
